# Inappropriate Sinus Tachycardia: Current Challenges and Future Directions

**DOI:** 10.19102/icrm.2018.090706

**Published:** 2018-07-15

**Authors:** Omar Z. Yasin, Vaibhav R. Vaidya, Shireen R. Chacko, Samuel J. Asirvatham

**Affiliations:** ^1^Division of Cardiovascular Disease, Mayo Clinic, Rochester, MN, USA; ^2^Department of Pediatrics and Adolescent Medicine, Mayo Clinic, Rochester, MN, USA; ^3^Christian Medical College, Vellore, India

**Keywords:** Ablation, inappropriate sinus tachycardia, laser ablation, sinoatrial node

## Introduction

Inappropriate sinus tachycardia (IST) is a perplexing disease that has an immense impact on the lives of patients suffering from the condition. To date, its cause remains a mystery, and whether the principal pathology even involves the sinoatrial (SA) node remains unknown. In no other area of rhythm management do we need to simultaneously assume the role of the cardiologist; the internist; the neurologist; and, in many instances, the psychiatrist. The severity of symptoms, in combination with our obvious lack of knowledge on how to relieve them, leads to great frustration and dissatisfaction for both patients and caregivers. At first glance, tantalizing tidbits of data suggest that IST tends to affect super athletes and psychologically traumatized individuals and has a gender predilection for women. These epidemiological data, combined with physiological data derived from various parts of the autonomic nervous system, seem to point us in a direction towards the truth. However, upon closer scrutiny, each of these directions lead to disparate paths. In this issue of *The Journal of Innovations in Cardiac Rhythm Management*, a potential new treatment option for IST is described.^21^ We took this opportunity to review the SA node anatomy and function in relation to what is currently known about IST and considered the limitations of current treatment modalities to highlight the potential role of the new treatment presented. We summarize prognostic data and existing treatment options so that we can clearly counsel our patients with IST to help alleviate their anxiety regarding issues such as left ventricular dysfunction and increased mortality and make them aware of the methods, albeit limited in number, that are available to help manage their symptoms so that they can lead normal lives.

Normal sinus rate and rhythm are the result of spontaneous depolarization of specialized pacemaker cells found in the SA node.^2^ The SA node is a linear, spindle-like structure located superiorly in the right atrium, near the junction of the atrium and the superior vena cava (SVC), beneath the epicardium.^2–4^ It is regulated by neurohormonal factors and controls the heart rate in response to changes in temperature, blood pressure, and sympathetic and vagal tones.^5,6^ At rest, the SA node fires at an intrinsic rate of 60 to 100 times per minute.^2^ A sinus rate of more than 100 beats per minute (bpm) is termed sinus tachycardia and is commonly a result of hyperadrenergic physiologic states, such as emotional stress and exercise, or an appropriate response to a pathologic state such as infection, fever, anemia, or hyperthyroidism.^7^ The latest American College of Cardiology/American Heart Association/Heart Rhythm Society guidelines define IST as symptomatic sinus tachycardia that is unexplained by physiological demands at rest, with minimal exertion, or during recovery.^7^ These symptoms (which are essential to make the diagnosis) are nonspecific and include weakness, fatigue, lightheadedness, and uncomfortable sensations such as a racing heart or palpitations.^6–8^ IST is a diagnosis of exclusion and can only be made after other etiologies for tachycardia have been ruled out, including postural orthostatic tachycardia syndrome, which often overlaps with IST.^5–7^

The prevalence of IST is estimated to be around 1% and prognosis is benign in terms of clinical outcomes and echocardiographic evidence of ventricular dysfunction.^7,9^ Nonetheless, its symptoms can be debilitating. The pathophysiology of IST remains incompletely understood at this time.^6,8^ Autonomic and neurohormonal dysfunction leading to inappropriately increased sympathetic tone or reduced parasympathetic tone have been implicated in the pathophysiology of IST.^10,11^ Other studies suggest a role for anti-β-adrenergic antibodies either directly causing tachycardia or which lead to sympathetic hypersensitivity.^12,13^ Finally, certain research has indicated that the accelerated rate of the sinus node could be due to intrinsic sinus node dysfunction, such as a channelopathy, rather than a response to extraneous factors.^14^ The treatment of IST aims to alleviate its symptoms, which is difficult to achieve given the nonspecific nature of its presentation and high prevalence of superimposed illnesses such as anxiety.^6^ Decreasing the heart rate does not necessarily alleviate the symptoms.^6,7^ Treatment approaches to IST center on reducing the sinus rate using lifestyle modifications, pharmacotherapy, or catheter ablation.^7,8,15–27^

Recent data support the role of exercise therapy in patients with IST.^15^ Pharmacotherapy for IST aims to reduce the heart rate by targeting the hyperpolarization-activated cyclic nucleotide-gated channel, β-adrenergic receptors, or calcium channels. Randomized controlled trials for the treatment of IST, which were only conducted for ivabradine, a drug that blocks the hyperpolarization-activated cyclic nucleotide-gated channel and inhibits the I(f) current, found a modest benefit (class IIa recommendation).^7,16–18^ Other medications include β-blockers or calcium channel blockers, but these are thought to be of limited benefit secondary to their side effects profile and the fact that their use is based on nonrandomized studies (class IIb recommendation).^7,19,20^

When conservative methods fail, invasive procedures can be considered for symptom control such as ablation or even stellate ganglion block.^21^ Several nonrandomized studies assessed the efficacy of radiofrequency ablation with variable degrees of success.^7,22^ Initially, complete sinus node ablation resulted in a high requirement for pacemaker implantation; thus, a subsequent less-aggressive approach of “sinus node modification” was considered, aimed at achieving a 25% reduction in the heart rate and a change in the P-wave morphology.^23–25^ Technical approaches included mapping and ablating the earliest site of atrial activation and/or ablating the SVC–right atrial appendage–crista terminalis confluence as visualized on intracardiac echocardiography. Ablation near the arcuate ridge also resulted in successful outcomes.^26^ A dual approach with endocardial and epicardial access can be useful, since the SA node is a subepicardial structure and epicardial access allows for the placement of a balloon to lift the phrenic nerve off the region of the SA node.^27^

Various energy sources are available for the ablation of cardiac tissue, including radiofrequency, cryo, microwave, and light amplification by stimulated emission of radiation (laser).^28^ The intrinsic properties of routinely used ablation modalities such as radiofrequency and cryoablation limit their role in the ablation of epicardial structures such as the SA node. In radiofrequency ablation, alternating current is directly applied to the myocardium, which causes heating at the electrode–tissue interface.^28^ This results in energy being directly delivered to the first millimeter or so of the cardiac tissue at the electrode–tissue interface, beyond which successful ablation requires longer application times or increased energy levels to ensure the conduction of thermal energy deeper into the myocardium to the targeted site.^28^ The farther the ablation target is from the electrode, the more complicated and unpredictable thermodynamics become, as energy dissipation occurs radially.^29,30^ Therefore, endocardial radiofrequency ablation of epicardial structures requires the creation of a transmural lesion and is limited by uncertainty regarding the depth of the lesion and increased temperatures at the electrode–tissue interface. The additional limitations of radiofrequency energy for sinus node modification include a risk of permanent sinus node damage, necessitating pacemaker implantation; SVC stenosis; phrenic nerve injury; recurrence of symptoms of inappropriate sinus tachycardia; and sinus tachycardia.^31–33^ To overcome these barriers, performing epicardial rather than endocardial radiofrequency ablation might provide a solution. Such can allow the delivery of energy to the epicardial aspect of the sinus node and provide phrenic nerve protection by inflation of a balloon to lift the phrenic nerve off of the right atrium. However, published experience with epicardial sinus node modification is limited and epicardial access has its own potential complications such as tamponade and pericarditis.

Cryoablation, which uses cooling to create irreversible tissue damage, has an advantage over radiofrequency ablation in that it forms a more discrete lesion, leads to less thrombosis, and causes less epithelial injury, making it less prone to cause collateral damage to surrounding structures.^28,34^ Despite the different approach employed, creating deep lesions via cryoablation is still limited, as the periphery of the cryolesion is not cooled to the same extent as the catheter–tissue contact point.^35^ Clinical experience with cryoablation for sinus node modification is very limited. There is a published case report in existence that employed cryoablation for sinus node modification that resulted in phrenic nerve injury.^36^

Limitations of currently used modalities can potentially be overcome by using electromagnetic energy, which theoretically allows for the creation of controlled, deep lesions in the myocardium. Microwave energy exists in the electromagnetic spectrum between 0.3 GHz and 300 GHz, and results in the oscillation of dipolar molecules such as water, which induces frictional heating and myocardial tissue damage.^28,29^ Unlike the previously described modalities, microwave energy propagation, not being limited by electrode/antenna–tissue contact, can more easily penetrate into deeper tissues.^28^ Microwave energy is used intraoperatively during surgical MAZE procedures.^37^ However, the design of microwave antennae is technically challenging, and there are no clinically available endovascular microwave catheters in existence at present. Studies are underway to evaluate the efficacy of microwave ablation for atrial flutter.^28,29,38^

Laser uses photons at specific wavelengths within the infrared, visible, or ultraviolet ranges of the electromagnetic spectrum, resulting in the heating of cardiac tissue by absorption of photons and the photothermal effect.^28^ Depending on the system used, different wavelengths of photons can be generated, which directly relates to the extent of tissue penetration and scatter. Laser has shown promise in the development of transmyocardial lesions, the depth of which were related to the duration of the photon energy application.^28,29,39^ Although there are no clinical experiences published, to our knowedge, using laser energy for sinus node modification, prior animal studies have evaluated the utility of neodymium-doped yttrium aluminum garnet laser for this purpose.^40,41^

The recent publication by Weber et al. is an excellent example of translating basic science research into direct patient care.^1^ The first part of this study demonstrates the feasibility of using laser ablation to create contiguous transmural lesions in five dogs. The authors used an open-irrigated 8-French tripolar catheter with 2 mm interelectrode spacing (for high-density mapping) that was capable of delivering up to 30 watts of power using a 1,064 nm diode laser. The team identified the site of the SA node using fluoroscopy and by monitoring local electrical potentials to identify the site of earliest atrial activation. Three laser applications were performed at the targeted site and ablation was confirmed by abolishing the local atrial potentials, which also resulted in a change in sinus cycle length. Postprocedure, the authors demonstrated a decrease in the mean heart rate for the animals and, at three months after the procedure, pathology samples confirmed the formation of ablation lesions, which nicely suggests the long-term success of the laser ablation.

The authors then presented a case report of a patient with IST treated with laser ablation. The procedure was successful in identifying a targeted site for SA node ablation and laser energy was successfully delivered without complication. However, unlike in the animal studies, the local atrial potentials were not abolished, though they did decrease. Other parameters such as cycle length before and after ablation were not reported, but do not seem to have significantly changed in the authors’ **Figure 7**.^1^ The patient developed palpitations postablation with orciprenaline administration at a heart rate of 95 bpm, but she did not experience anxiety, which she had previously demonstrated with use of this medication. During the 4.9 years of follow-up, the patient remained asymptomatic with normal heart rates at rest and during exercise, despite having heart rates of up to 110 bpm on Holter monitor soon after IST ablation. The study demonstrated the successful identification of a target site for ablation in humans and the successful application of laser energy without complications. However, the inherent limitations of the study design (ie, it’s a case report) limits the ability to make conclusive statements about the success of the procedure in patients with IST.

As the authors clearly identified, future direction could capitalize on this proof-of-concept study to recruit more patients with IST to assess the success of using laser energy for SA node ablation. This would provide more information about the efficacy and safety of using laser energy for the treatment of IST in humans. Future investigations would need to identify clear inclusion criteria, since the diagnosis of IST, based on current guidelines, remains vague. Although challenging from a trial design standpoint, use of a sham-control arm would add to the scientific rigor of any future ablation studies on IST. Outcome measures also need to be clearly defined, which will be difficult to quantify given that treatment goals are often subjective and symptoms are nonspecific. The ideal ablation modality for IST should incorporate accurate mapping of the sinus node and ablation of the sinus node regions such that chronotropic reserve is preserved, while avoiding adverse effects from ablating nearby critical structures. Central and peripheral autonomic hyperactivity could drive IST. Autonomic modulation can reduce the sympathetic tone and has a role in the treatment of refractory ventricular tachyarrhythmias.^42^ Autonomic modulation is being actively studied as a modality for the treatment of atrial arrhythmias, hypertension, and episodic hypotension.^43,44^ Autonomic modulation could be effective in ameliorating IST in the future, without direct ablation performed in the sinus node region.

In conclusion, the diagnosis and treatment of IST remains challenging. It is a diagnosis of exclusion and is characterized by a symptomatic sinus rate > 100 bpm without a physiologic demand. Its pathophysiology is unclear, and current theories suggest a component of intrinsic SA node dysfunction, autoantibodies, or increased sympathetic tone. While data remain limited, treatment modalities such as lifestyle modification, pharmacotherapy, and catheter ablation have potential benefit. Preliminary results suggest that laser modulation of the sinus node holds promise for the treatment of IST.
